# Sero-Epidemiology and Associated Risk Factors of Foot-and-Mouth Disease (FMD) in the Northern Border Regions of Pakistan

**DOI:** 10.3390/vetsci10050356

**Published:** 2023-05-16

**Authors:** Munib Ullah, Yanmin Li, Kainat Munib, Hanif Ur Rahman, Zhidong Zhang

**Affiliations:** 1 State Key Laboratory of Veterinary Etiological Biology, Lanzhou Veterinary Research Institute, Chinese Academy of Agricultural Sciences, Lanzhou 730030, China; drmunib15@gmail.com; 2 Department of Clinical Studies, Faculty of Veterinary and Animal Sciences, PMAS-Arid Agriculture University Rawalpindi, Rawalpindi 10370, Pakistan; 3 College of Animal Husbandry and Veterinary Medicine, Southwest Minzu University, Chengdu 610041, China; 4 Department of Sociology, Allama Iqbal Open University Islamabad, Islamabad 44310, Pakistan; cscuniverse323@gmail.com; 5 Virology Section, Centre of Microbiology and Biotechnology (CMB), Veterinary Research Institute Peshawar, Peshawar 25130, Pakistan; drhanif001tanha@gmail.com

**Keywords:** FMD, FMDV, sero-epidemiology, risk factors, northern Pakistan

## Abstract

**Simple Summary:**

To investigate seroprevalence and identify the risk factors associated with foot-and-mouth disease (FMD) in Pakistan’s northern border region, 385 serum samples were compiled from 75 flocks/herds in seven sub-regions in this region, including sheep (*n* = 97), goats (*n* = 142), cattle (*n* = 60), and buffaloes (*n* = 86) and tested using an FMDV 3ABC-Mab-bELISA for detection of antibody to FMD virus non-structural protein. The results showed that an overall apparent seroprevalence in this region was 67.0% of the total analyzed samples, the seroprevalence in sheep, goats, cattle, and buffaloes were 51.5%, 71.8%, 58.3%, and 82.5%, respectively. From the different risk factors investigated, age, sex, species of animal, seasons, flock/herd size, farming methods, outbreak location, and nomadic animal movement in search of water and pasture from region to region were found to be significantly associated (*p* < 0.05) with the seroprevalence of FMD. FMD remains a significant disease of ruminants in Pakistan’s northern border region. Sero-epidemiology and identification of associated risk factors of FMD is essential to bind the consequence of FMD in the region.

**Abstract:**

The present cross-sectional survey was carried out to investigate the distribution and risk factors of FMD in Pakistan’s northern border regions. About 385 serum samples were compiled from small ruminants (239) and large ruminants (146) and tested using 3ABC-Mab-bELISA. An overall apparent seroprevalence of 67.0% was documented. The highest seroprevalence of 81.1% was reported in the Swat, followed by 76.6% in Mohmand, 72.7% in Gilgit, 65.6% in Shangla, 63.4% in Bajaur, 46.6% in Chitral and lowest 46.5% in Khyber region. Statistically significant variations in seroprevalence of 51.5%, 71.8%, 58.3%, and 74.4% were recorded in sheep, goats, cattle, and buffaloes, respectively. From the different risk factors investigated, age, sex, species of animal, seasons, flock/herd size, farming methods, outbreak location, and nomadic animal movement were found to be significantly associated (*p* < 0.05) with the seroprevalence of FMD. It was concluded that proper epidemiological study, risk-based FMD surveillance in small ruminants, vaccination strategy, control measures for transboundary animal movement, collaborations, and awareness programs need to be practiced in the study regions to investigate the newly circulating virus strains in large and small ruminants and associated factors for the wide seroprevalence to plan proper control policies to bound the consequence of FMD in the region.

## 1. Introduction

Foot and mouth disease (FMD) is a major transboundary, acute, highly communicable, and economically important viral disease of all cloven-footed animals and wild ruminants [1]. FMD severely affects livestock production and trade associated with animals and animals’ products regionally and globally. The global FMD control strategy was validated in 2012 to reduce the FMDV load in enzootic situations and to sustain the FMD-free status of different countries [2]. The disease is caused by the foot and mouth disease virus (FMDV). The FMDV is categorized into the family *Picornaviridae* and the genus *Apthovirus*. This virus is the smallest non-enveloped virus having ten non-structural and four structural proteins. The FMDV has seven serotypes, and each of the serotypes has further antigenic and epidemiological-based distinct subtypes. The extensive variation is due to the great frequency of mutation, genetic recombination, and quasi-species phenomenon [3,4,5]. As per World Organization for Animal Health (WOAH), FMD is a disease that needs to be immediately reported and properly investigated on time to stop its spread in the region [2]. FMD is characterized by high fever, the development of erosions, and vesicles in the mouth resulting in saliva driveling. Vesicles also appear on other parts of the animal body, including the teats, nose, and feet. When vesicles on the feet rupture, the affected animals often feel reluctant to stand and move [6,7]. The disease causes weight loss in growing adults and significantly declines milk production in dairy animals [8]. The disease may cause high mortality in young stocks due to cardiac arrest succeeding myocarditis [9]. FMD could persist in goats and sheep for up to nine months [10]. The clinical differential diagnosis of FMD from other vesicular diseases is difficult [6]. So, laboratory confirmation of FMD-suspected cases is mandatory. The differentiation between infected and vaccinated animals (DIVA) protocol is of great importance to detect the antibodies against the non-structural proteins (NSPs) of FMDV. Using a highly sensitive and specific 3ABC competition antibody, ELISA can provide same-day results and be useful for FMD routine general screening [11,12].

FMD is enzootic in Pakistan and causes gigantic economic damage to commercial livestock producers [13]. As per the report of the Food and Agriculture Organization (FAO), there are no proper strategies for vaccination, a supply chain of vaccines to animal production systems, and the indigenous marketplace is full of unrestrained vaccines of suspicious efficacy [14]. Furthermore, FMD is enzootic with serotypes A, Asia 1, and O in neighboring countries, including Tajikistan, India, Bangladesh, China, Nepal, Iran, Kazakhstan, Kyrgyzstan, Turkmenistan, and Afghanistan. Some of these states share boundaries with Pakistan, and there is a lot of cross-border animal movement between them [15,16,17,18,19,20,21], and these serotypes are a sustained threat to industry and local farmers in Pakistan. Pakistan faces major challenges to controlling FMD the inappropriate diagnostic resources and lack of constant genotypic surveillance of FMD, difficulties in monitoring the vaccine market, the lack of awareness of fundamental biosecurity measures, and restrictions on animal movements at local, trans-provincial, and international borders.

The intrinsic associated risk factors of FMD sero-positivity are the age of the animal, sex, general health condition, and animal breed in the region, while livestock farming system (intensive, semi-intensive, and extensive), herds/flocks configuration, size of herds/flocks, history of regional animal movement, livestock wildlife interface in the region, outbreak location, awareness of local farming communities regarding FMD, the agroecological status of the target region, and communal grazing and watering practices are considered as extrinsic associated risk factors of FMD sero-positivity [22,23]. Furthermore, in the annual spiritual festival of Eid Quraban (Eid al-Adha), a gigantic unrestricted animal movement takes place between various provinces of Pakistan, as well as with Afghanistan and adjacent neighboring regions, where thousands of animals are transported and act as contributing factors for FMD positivity while carrying the virus from hyperendemic territories to the study region and vice versa. Regionally including Pakistan, different studies have been conducted in large ruminants, but inadequate reports exist on FMD seroepidemiology using 3ABC-Mab-bELISA and related risk factors in large as well as small ruminants. That is why the current epidemiological investigation was carried out to estimate the seroprevalence, identify the associated risk factors and hotspot trends of foot and mouth disease in rarely investigated regions of Pakistan’s northern border, and will identify where, how, and when intensive surveillance and immunization along with biosecurity procedures essential to be employed for the control of the infection from the research area and adjacent neighbor countries in consonance with the FMD Global Control Strategy.

## 2. Materials and Methods

### 2.1. Research Area

**Figure 1:**
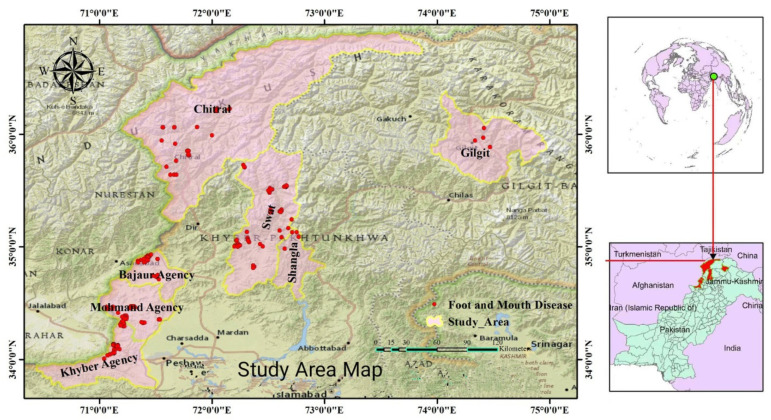
Hotspot map of the spatial distribution of FMD.

The study was conducted on Pakistan’s northern border, adjacent to Afghanistan, China, and Tajikistan borders (Figure 1). The targeted districts in this region include Swat (35°12′ N 72°29′ E), Shangla (34°52′ N 72°39′ E), Chitral (35°50′ N 71°47′ E), Bajaur agency (34°41′ N 71°30′ E), Khyber agency (32°40′ N 69°51′ E), Mohmand agency (34°30′ N 71°20′ E), and Gilgit region (35°55′ N 74°18′ E). The first three districts belong to the formerly provincially administrated tribal areas (PATA) of Pakistan. The provincially administrated tribal areas (PATA) were the former administrative subdivision of Pakistan designated in Article 246(b) of the constitution of Pakistan. The remaining three agencies (Bajaur, Khyber & Mohmand) belong to the formerly federally administrated tribal areas (FATA). The FATA was a semi-autonomous tribal region in northwestern Pakistan, which existed from 1947 until being merged controversially with the neighboring province of Khyber Pakhtunkhwa in 2018. Furthermore, Gilgit is the capital city of Gilgit–Baltistan (GB). GB borders Azad Kashmir to the south, the province of Khyber Pakhtunkhwa to the west, the Wakhan Corridor of Afghanistan to the north, the Xinjiang region of China to the east and northeast, and the Indian-administered union territories Jammu and Kashmir and Ladakh to the southeast. These conflict-hit territories were declared as neglected areas for monitoring and epidemiological investigation of endemic diseases, including FMD.

### 2.2. Study Animals

The study animals consisted of cattle, buffaloes, sheep, and goats in seven selected districts, and the study units were unvaccinated animals that were more than six months. Sheep and goats which are adapted to the study areas are classified under indigenous and cross breed, while cattle were of Achai, Sahiwal, and cross breed, on the other hand, buffalo were of Azakheli, Kundi, Nili-Ravi, and cross breed.

### 2.3. Study Design and Sampling Strategy

A cross-sectional study design with a multistage cluster sampling technique was implemented in this investigation with hierarchical stages (province, division, district, tehsil, and grazing area or village) to reach the sampling units. Clustering was prepared on the tehsils level for various villages based. A single-level cluster of animals was considered a village that shared mutual feeding and watering areas. Two regions were selected purposively based on sheep and goat populations and different agroecological aspects, whereas the remaining districts were randomly selected. Fifteen tehsils and a total of seventy-five flocks/herds were selected conveniently based on road access, from which 5–10 animals were sampled randomly from willing owners. The sample size was calculated on the formula described by Thrusfield, M. [24]. The formula is *n* = 1.96^2^ Pexp (1−Pexp)/d^2^ where *n* (sample size); d (desired absolute precision), and Pexp is (expected prevalence).

### 2.4. Sample Collection Procedures

Blood samples (about 5 mL) were collected directly from the jugular vein of animals by venipuncture using a sterile syringe and needles and then transferred to a labeled gel-barrier tube with an identification code. After centrifugation, serum was collected. A clear serum was poured into an Eppendorf tube (2 mL) and categorized consequently, and stored in the freezer till its arrival at the laboratory and stored in a freezer (−20 °C). All samples were transported to the Veterinary Research Institute, Peshawar, Pakistan, by keeping the cold chain and stored at −20 °C.

### 2.5. FMDV 3ABC-ELISA

3ABC-Mab-bELISA kit was used to detect antibodies against 3ABC proteins of FMDV in sera according to the instruction of Fu et al. [25]. The percentage inhibition (PI) of the controls and the test sera was calculated using the formula below. The OD450 values of all samples are expressed as percentage inhibition (PI) relative to the OD450 max. PI = (1-OD450 test sample/OD450 max) × 100%. The experiment was only tenable when OD450 max > 1.000, the mean percentage inhibition of the weak positive control > 50%, and the mean percentage inhibition of the positive control > 70%. When the PI ≥ 50%, the serum was positive; when the PI < 50%, the serum was negative.

### 2.6. Data Collection

A structured questionnaire was projected to collect data about animal species, sex, age, flock size, grazing dynamics, farming method, vaccination status, the entry of new animals into a herd/flock, information about the outbreak of FMD, nomadism & cross-boundary movements, returned of unsold animals into the flock/herd, the appearance of the clinical sign of FMD and availability of nearby veterinary services. The geographical positioning system (GPS) coordinates were obtained in the form of latitudes and longitudes. The size of the flock/herd was categorized as less than 50, 51–100, 101–200, 201–300, 301–500, and more than 500. The age of animals was noted through physical observation, dentition, and inquiring from the farmers. The age was then classified as less than 1 year, 1 year, 2 years, 3 years, and above 3 years.

### 2.7. Data Management and Analysis

The MS Excel spreadsheet 2020 program was used for data entry, and the data were coded and transferred to Statistical Package for Social Sciences (SPSS) version 20 (SPSS Inc., Chicago, IL, USA). Overall apparent seroprevalence was calculated by Thrusfield, M. [26]. The formula is given by: Apparent prevalence = (positive samples/total number of animals sampled) × 100. The Epi-Info online software (version 3.5.1) was used to calculate the confidence interval for proportions. Univariable logistic regression analysis for the proportions was carried out with *p* = 0.25. This was verified additionally by multivariable logistic regression analysis for the decision with a probability predictive limit of less than 5%. The odds ratio (OR) was used to associate the statistical power of FMD positivity with various possible risk factors. The interaction consequence of significant risk dynamics in the multivariable logistic regression analysis was also assessed. Model fitness was calculated by applying the Hosmer-Lemeshow goodness of the test (*p*-value > 0.05). ArcGIS 10.8.1 was used to design hotspot maps from GPS coordinates to highlight the location of each case in their localities.

## 3. Results

### 3.1. Apparent Seroprevalence of FMD in the Northern Border Regions of Pakistan

A total of 385 serum samples from 75 flocks/herds in seven districts were collected and tested using a foot-and-mouth disease non-structural protein 3ABC-Mab-bELISA. Based on the detection rate of the FMDV antibody, overall apparent seroprevalence of 67.0% (95% CI = 64.0–70.9%) was recorded in the target region.

The district-wise seroprevalence of FMD was then analyzed and shown in Table 1. The highest seroprevalence of 81.1% (95% CI = 72.4–89.4%) was recorded in the Swat district, followed by 76.6% (95% CI = 65.9–87.3%) in Mohmand Agency, 72.7% (95% CI = 59.5–85.8%) in Gilgit, 65.6% (95% CI = 49.7–79.0%)63.4% (95% CI = 48.6–78.1%) in Bajaur Agency, 46.6% (95% CI = 32.0–61.2%) in Chitral (35°50′N 71°47′E), and lowest 46.5 (95% CI = 31.6–61.4%) in Khyber Agency.

For species-wise seroprevalence of FMD (Table 1), an apparent seroprevalence of 51.5% (41.6–61.4%), 71.8% (64.4–79.2%), 58.3% (45.8–70.8%) and 82.5% (68.0–89.7%) was recorded in sheep, goats, cattle and buffaloes, respectively. The highest seroprevalence was observed in buffaloes and the lowest in the sheep population.

### 3.2. Risk Factors Associated with FMD Seropositivity

Univariable logistic regression was used to analyze risk factors associated with FMD positivity in sheep, goats, cattle, and buffaloes. Various factors omitted from the model by applying univariable logistic regression analyses with a *p*-value of 0.25 were vaccination status, an outbreak of FMD or FMD-affected animals in the area in the last 15 days, introducing new animals, return of unsold animals from the market, and type of flock/herd. According to univariable logistic regression analysis, seasons, herd/flock size, species of animal, sex, age, outbreak location, farming methods, and nomadic animal’s movement in search of grazing points and water were found to significantly connected risk factors (*p* < 0.05) with FMD seropositivity in the region (Table 1).

### 3.3. Hotspots of the Spatial Distribution of FMD

On spatial epidemiological analysis of outbreak data, FMD risk hotspots indicated a wide deviation in the various regions of northwestern and northeastern Pakistan at different periods. Most of the study regions were considered neglected areas of disease investigation, including FMD. Therefore, special attention was taken to monitoring the nomadic flock/herds and participating in study activities in harsh full conditions. Figure 1 shows the spatial distribution of FMD in selected neglected areas of Pakistan’s northern border regions based on disease coordinates (latitudes and longitudes). The seven FMD disease hotspots trend categories were identified across different sub-regions in Pakistan’s northern border region based on both the detection rate of FMDV antibody. The greenish zones represent study areas, while the yellow spots show the burden of FMD cases in different study districts. These disease hotspots were identified through the tools of participatory epidemiological assessment, questionnaire-based surveys, evidence of traditional routes of transboundary animal movements to these areas, seasonal migration within the country and across the border to these regions, livestock practices without vaccination, and based on current results, which shows that the disease is prevalent in these regions and act as a continue spreading points locally and regionally. FMD disease hotspots trend categories across different sub-regions in Pakistan’s northern border region based on the detection rate of FMDV antibodies are shown in Figure 1.

The map identified seven FMD disease hotspots trend categories across different sub-regions in Pakistan’s northern border region. The greenish zones encircled by red boundaries represent study areas, while the yellow spots show the burden of FMD cases in different study districts (Swat, Chitral, Gilgit, Shangla, Mohmand, Khyber, and Bajaur). The map was created using ArcGIS, 10.8.1 while dragging GPS coordinates to the page and dropping it on the map drop zone the authors.

## 4. Discussion

Although a wide variety of studies have been taken in the domain of the FMD world widely, the current study provides a spark to FAO/WOAH for the achievement of a global FMD control strategy in the future by understanding the epidemiology and controlling the disease in the previously neglected or conflict-hit territories where the evidence of small ruminants–large ruminants and livestock-wildlife interfaces exist. The presence of the FMDV virus in these regions was demonstrated by clinical picture and by using FMDV 3ABC-Mab-bELISA. Based on the result of the current serological survey, FMD remains a significant disease of ruminants in Pakistan’s northern border region, with an overall apparent seroprevalence of 67.0%. The seroprevalence of FMD in this region was higher than what has been documented in other parts of the country [26,27,28,29] and also higher than that investigated in Israel, Sudan, Libya, and Ethiopia [30,31,32,33]. The dissimilarities in the FMD seropositivity found in neighboring states paralleled to the current study area might also be attributed to differences in livestock supervision systems, seasonal differences, altered host population density, topographical dissimilarities, disease control programs, sampling techniques used or technical knowledge levels of natural immunity and variable natural FMDV infection rates in various geographical zones.

Deviations of FMD seroprevalence in terms of spatial distributions have been recorded throughout the study area. The higher seroprevalence of FMD was in Swat, followed by Mohmand Agency, Gilgit, and the lowest in Khyber Agency. These may be due to intensive unrestricted cross-boundary movements of unvaccinated herds/flocks between these regions with the neighboring countries where FMD outbreaks have been reported in the past. In addition, negligence in vaccination due to poor livestock supervision and the practice of joint grazing structures, and massive nomadism might be contributing factors to this higher seroprevalence in the study area. The interface amongst livestock, domesticated yaks, and small wild ruminants in pastoralism, especially in regions such as Gilgit and some hotspots of Swat Valley, might also have to contribute risk factors to the greater FMD seroprevalence. The high FMD seroprevalence in Swat, Gilgit, and Mohmand agencies might also be correlated to the deviations in climatic dynamics.

In the finding of the current study, the flock or herd size was considerably linked with the FMD seroprevalence. While keeping the other elements persistent, animals from medium (201–300) and large flock/herd size (301–500) were 1.5 and 1.6 times more seropositive as paralleled to those individuals belonging to small herd size, respectively. The findings of our investigation were in line with [34,35,36], who studied that there was a positive correlation between FMD seropositivity and the size of the herd or flock. This may be associated with the transmission dynamic of FMD and its infectious nature, which is correlated to the gathering of large and small ruminants, leading to enhance the rate of direct interface and therefore increasing the probability of FMD transmission among individuals and herds or flocks.

The current study provides evidence that the gender-wise FMDV NSP seropositivity was significantly more in females 98.7% than in males 17.8%. A consistent report was documented in some parts of Punjab Province, with 26.26% seroprevalence in female and 11.46% seroprevalence in male animals [26]. The outcomes of current investigations are also inconsistent with those of Awan et al. [37], Megersa et al. [38], and Mohamoud et al. [39]. However, the outcomes of one Ethiopian study are different from the current findings, where they recorded 8.27% seropositivity of FMD in females, which is less than 15.7% seropositivity in male animals [36]. Furthermore, in contrast to the findings of both the current and other studies conducted in the Ethiopian region, there is a record that showed no inconsistency in the associated risk with FMD spread among the female and male populations [40]. Both genders, female and male, have an equal probability of being infected by FMDV. Though, the dissimilarity in infection conditions might be due to the long raising of females as paralleled to male animals, which are sold at an earlier stage of age. Another conceivable clarification for this dissimilarity might be the physiological strain felt by female stocks due to gestation, lactation, and diet.

A significant change was detected in the seroprevalence of FMD between matured (98.1%) and young animals (1.7%). This finding is in agreement with the results of others [41,42]. The outcomes of the present serosurvey are also in line with the findings of the former studies of Gelaye et al. [36], Awan et al. [37], Megersa et al. [38], Mohamoud et al. [39], Mannan et al. [43] and Chepkwony et al. [44]. Gelaye et al. [36] documented a non-significant relationship between FMD seropositivity and animal age in the Ethiopian region, which is in contrast to the findings of the current study. The lower seropositivity of FMD in younger stocks might be due to the lower contact rate and insistent passive immunity. Whereas higher susceptibility in older animals may be due to malnourishment, deprived management structure, weak immunity, or might be due to the perspective that older stocks might have greater vulnerability to FMDV at pesters, watering hotspots and at the marketplace as compared to age groups less than 1 year. Consequently, mature animals may have developed an infection from various serotypes and strains; therefore, generating antibodies in contradiction of numerous viruses spread FMD. It may also associate with collective seropositivity through repetitive infection in their long lifespan.

Animals raised in a communal system (nomadism) were probable to have greater seroprevalence with corresponding seroprevalence levels of 98.0%, while low seroprevalence was recorded in the animals raised in other farming systems such as sedentary, transhumance, and mixed or intensive systems. These findings were in line with the outcomes of Balinda et al. [45], who investigated that the cause of the infection and spread of FMD was the closed interface among livestock in communal grazing and joint housing. Though, the finding of the present investigation is contrary to the outcomes of Chepkwony et al. [46], who investigated that, only mixed production systems indicated a statistically positive relationship with FMD seropositivity, while other production systems, including nomadism and transhumance, showed a significant negative association with FMD seropositivity. It might be due to sampling size, farming method, animal management system, climate factors, and study area differences.

Statistically significant variation in seroprevalence in the different species of ruminants was recorded. Of the total analyzed samples, the highest seroprevalence was observed in buffaloes, followed by goats and cattle population, and the lowest in the sheep population. Bayissa et al. [35], Megersa et al. [38], Jenbere et al. [40], Beyene et al. [47], and Mesfine et al. [30] also documented prominently greater seropositivity of FMD in large ruminants predominantly in cattle than in small ruminants in the west Ethiopian region. Typically, higher serological prevalences in pastoral regions were documented in different studies. This might be due to greater animal movement in pastoral production; this type of production system facilitates a higher interaction rate and the transmission of the infection. Further, this indicates that a mixed type of animal production system where different species are reared together is the main cause of FMD spread among large ruminants. In the current survey, high seroprevalence was documented in goats (71.8%) as compared to sheep (51.1%). The outcomes of the current study are inconsistence with the findings of the Nigerian study, where a greater seroprevalence of FMD, 15%, was recorded in goats and 9.3% in sheep [48]. The present study is also in line with the study compiled in the Indian region [49]. However, our findings are in contrast to one study conducted in Pakistan, where 19.44% seropositivity was recorded in goats and 21.27% was recorded in sheep [12], and a Nigerian study, where a higher seroprevalence of FMD (41.66%) was recorded in sheep paralleled to goats (21.8%) [50]. Similarly, the findings of a study conducted in Jordan are in contrast with the current study, where a higher seroprevalence of FMD (10.4%) was documented in sheep paralleled to goats (6.3%) [51].

Associated risk factors that were statistically non-significant with seropositivity of FMD in the region were an outbreak of FMD or FMD-affected animals in the area in the last 15 days (*p* > 0.05), the introduction of new animals into the flock/herd in the last 15 days (*p* > 0.05), type of flock/herd (*p* > 0.05), return of unsold animals from the market (*p* > 0.05). However, the appearance of clinical signs of FMD and seasons were significantly associated with the seroprevalence of FMD in the study region (*p* < 0.05) (Table 1). The highest seroprevalence was recorded in the monsoon season (August–September). During this season, the humidity and temperature rise, and the incidence of FMD cases also rises with it. These findings are in line with the outcomes of Dion et al. [52]. The higher frequency of FMD infection in hot moist seasons is due to the appropriate eco-friendly circumstances for the insistence and propagation of FMDV, as retrospectively documented by [53]. The persistence of airborne viruses is sustained by high-flown moisture [54].

## 5. Conclusions

An overall apparent FMD seroprevalence of 67.0% was documented in sheep, goats, cattle and buffaloes in Pakistan’s northern border regions, indicating that FMD remains a significant disease of ruminants in this region. Furthermore, age, sex, species of animal, flock/herd size, farming methods, seasons, outbreak locations, nomadism, and transboundary animal movement in seek of grassland and water was detected as significantly related (*p* < 0.05) with FMD seroprevalence. The present epidemiological investigation predicted seven FMD hotspots across different sub-regions in this region. To better understand the transmission dynamic of FMD in the region, further large-scale epidemiological investigations are required, which would help prevent the spread of the FMD virus in the region and contribute to the FMD Global Control Strategy.

## Figures and Tables

**Table 1:** Overall univariable logistic regression analysis of risk factors for FMD seropositivity.

Variables	Cluster	Tested Samples	Positives	Prevalence in % (95% CI)	Odds Ratio (95% CI)	*p*-Value
Species	Sheep	97	50	51.5 (41.6–61.4)	1.769 (1.296–2.416)	0.002
	Goat Cattle Buffalo (ref)	142 60 86	102 35 71	71.8 (64.4–79.2) 58.3 (45.8–70.8) 82.5 (68.0–89.7)		
Age	<1 year 1 year	117 6	02 00	1.7 (0.0–4.05) 0.0 (0.0–0.0)	0.655 (0.470–0.914) 0.000 (0.00–0.00)	0.000
	2 year	30	30	100 (100–100)		
	3 year >3 year (ref)	158 74	155 71	98.1 (96.2–100) 95.9 (90.0–100)		
Sex	Male	151	27	17.8 (7.6–21.8)	0.621 (0.491–0.985)	0.000
	Female (ref)	234	231	98.7 (98.0–100)		
Flock/Herd size	<than50 51–100	116 10	1 5	0.86 (0.0–2.5) 50.0 (19.0–80.9)	0.781 (0.053–4.4) 1.336 (0.187–11.753)	
	101–200	20	15	75.0 (56.0–93.9)	1.213 (0.158–5.532)	0.000
	201–300 301–500 >than 500 (ref)	99 140 0	98 139 0	98.9 (97.6–100.0) 99.2 (98.0–100.0) 0.00 (0.0–0.0)	1.592 (0.215–11.766) 1.628 (0.225–11.966)	
Vaccination status	Non-vaccinated	385	258	67.0 (61.2–72.4)	1.201 (0.301–3.981)	0.987
Outbreak of FMD or FMD affected animals in the area in last 15 days	Yes No	128 257	85 173	66.4 (55.5–73.8) 67.3 (61.1–75.1)	0.901 (0.502–1.521)	0.254
Introduced new animals	Yes	141	97	68.7 (59.8–75.8)	0.983 (0.512–1.821)	0.591
	No (ref)	244	161	65.9 (59.1–70.6)		
Nomadic animals movement in the area in last 15 days	Yes No (ref)	253 132	248 10	98.0 (96.3–99.8) 7.5 (0.21–7.1)	0.701 (0.485–0.924)	0.002
Farming methods	Sedentary	127	13	10.2 (4.3–14.7)	2.990 (0.999–15.001)	0.001
	Transhumance	42	34	80.9 (63.3–91.8)		
	Nomadic Mixed (ref)	210 6	206 5	98.0 (96.3–100.0) 83.3 (53.5–100)		
Clinical sign of FMD	Yes No	145 240	129 129	88.9 (83.1–94.3) 53.7 (45.5–58.7)	0.071 (0.041–0.192)	0.000
Type of flock/herd	Sheep Goat Cattle Buffalo Mixed (ref)	72 93 36 54 130	41 63 24 50 80	56.9 (45.5–68.3) 67.7 (58.2–77.2) 66.6 (51.2–82.0) 92.5 (73.9–99.2) 61.5 (53.1–69.9)	1.028 (0.378–2.800) 1.324 (0.498–3.519) 0.452 (0.125–1.643) 0.288 (0.058–1.420)	0.081
Return of unsold animals from the market	Yes No	50 335	37 221	74.0 (50.1–81.4) 65.9 (59.8–70.1)	1.612 (0.525–4.125)	0.745
Outbreak location	MohmandAgency Khyber Agency Bajuar Agency Swat Chitral Gilgit Shangla (ref)	60 43 41 85 45 44 67	46 20 26 69 21 32 44	76.6 (65.9–87.3) 46.5 (31.6–61.4) 63.4 (48.6–78.1) 81.1 (72.4–89.4) 46.6 (32.0–61.2) 72.7 (59.5–85.8) 65.6 (49.7–79.0)	0.751 (0.165–3.414) 4.918 (1.152–21.007) 1.881 (0.401–8.829) 3.073 (0.747–12.646) 0.837 (0.187–3.739) 0.487 (0.129–1.831)	0.016
Seasons Monsoon Post Monsoon Winter Winter Summer	Aug-Sep Oct-Nov Dec-Jan Feb-March April-June	48 25 171 117 24	40 12 115 78 13	83.3 (72.7–93.8) 48.0 (28.4–67.5) 67.2 (59.1–74.03) 66.6 (54.1–71.9) 54.1 (34.2–74.1)	0.158 (0.037–0.671) 0.436 (0.092–2.071) 0.783 (0.232–2.545) 0.495 (0.142–1.732)	0.020

## Data Availability

The processed/analyzed dataset during the current study is available from the corresponding author upon request.
